# The Dyadic Context of Preadolescent Girls’ Depressive Symptoms: Elucidating The Role of Biobehavioral Synchrony

**DOI:** 10.1007/s10826-026-03300-6

**Published:** 2026-04-10

**Authors:** Jennifer A. Somers, Elena Cannova, Gabrielle R. Rinne, Chengzhen Angelina Meng, Kyra Fisher, Yussof Khalilian, Gabrielle MacNaughton, Tiffany C. Ho, Steve S. Lee

**Affiliations:** 1 Department of Psychological Sciences, Auburn University, Auburn, AL USA; 2 Department of Psychology, University of California, Los Angeles, CA USA

**Keywords:** dyadic synchrony, emotion socialization, respiratory sinus arrhythmia, child depression, parental depression

## Abstract

Biobehavioral synchrony represents a dynamic interpersonal mechanism that influences parent and child depression risk via parent-child interactions, which may operate directly or in concert with depression-related impairments in parenting. We hypothesized that parent depressive symptoms and unsupportive emotion-related socialization behavior (ERSB) would negatively predict positive affect and physiological synchrony, and that these parent factors would moderate the effects of dyadic synchrony on daughters’ depressive symptoms. Eighty parent (87.5% female)-daughter (*M*_child age_ = 8.26 years, *SD*_child age_ = 1.51 years; 38.0% White) dyads participated in a 5-minute conflict discussion task, during which second-by-second estimates of parent unsupportive ERSB and parent and daughter affect and respiratory sinus arrhythmia (RSA) were obtained. Parent self-reported depressive symptoms and rated child behavior problems. Contrary to hypotheses, path models suggested that neither parent depressive symptoms nor unsupportive ERSB was associated with dyadic synchrony of positive affect or RSA. However, as expected, parent depressive symptoms and unsupportive ERSB moderated predictions of daughters’ depression symptoms from dyadic synchrony. The nature of these interactive effects depended on the synchrony modality (i.e., positive affect, RSA) and parent factor under investigation. RSA synchrony during parent-daughter conflict discussion buffered against the adverse effects of parent depressive symptoms on daughters. Importantly, these patterns were specific to daughters’ depressive symptoms rather than general dimensions of child psychopathology. These results underscore the centrality of multimodal, dynamic processes through which parent-daughter interactions may shape depression risk in daughters.

**Supplementary Information:**

The online version contains supplementary material available at 10.1007/s10826-026-03300-6.

## Highlights

Parents and their school-aged daughters vary in the extent to which they synchronize their positive affect and physiological functioning during a conflict discussion.

Positive affect and physiological synchrony were not related, highlighting unique processes of biobehavioral synchrony.

Further, both forms of synchrony modulated parent-related risk, but in different ways depending on the domain of synchrony and type of parent risk factor.

**Supplementary Information:**

The online version contains supplementary material available at 10.1007/s10826-026-03300-6.

Compared to boys, female adolescents are three times more likely to develop depression, which powerfully confers risk for diverse adverse outcomes across development (Daly et al., 2022; Fletcher, 2008; Hasler et al., 2005; Keenan-Miller et al., 2007; Lewinsohn et al., 1998). Even subclinical levels of youth depressive symptoms place girls at risk for future internalizing disorders and social and educational impairments throughout the lifespan (e.g., Bertha & Balázs, 2013; Johnson et al., 2009; Klein et al., 2009; Rohde et al., 2009; Shankman et al., 2009). Sex differences in internalizing problems emerge in childhood (Breslau et al., 2017), underscoring the urgent need to identify unique risk processes associated with early elevations in girls’ depressive symptoms. Interpersonal contextual adversities, including exposure to clinical and subclinical parental depressive symptoms as well as unsupportive parenting, are among the most robust risk factors for adolescent depression (Goodman, 2020; Gotlib et al., 2023; Natsuaki et al., 2014). During middle childhood especially, parents affect the development of children’s emotion self-regulation and socioemotional competence through supportive reactions to children’s emotions (i.e., supportive emotion-related socialization behaviors). This not only consists of acknowledging and labeling children’s emotions but also coaching children’s problem-solving and regulatory strategy use (Eisenberg et al., 1998; Morris et al., 2007). Precipitous increases in family conflict and emotion variability during the transition to adolescence provide parents with repeated opportunities to respond to their child’s emotions in supportive ways that facilitate internalization of appropriate affect display rules, greater emotional awareness and understanding, and support joint problem-solving (Branje, 2020; Collins et al., 2002; Larson et al., 2002). By contrast, *unsupportive* emotion-related socialization behaviors (ERSBs; e.g., minimizing or punishing children’s emotions; inhibiting youth autonomy) are more common in families affected by clinical and subclinical parental depressive symptoms(Choi et al., 2023; Labella et al., 2021; Lins et al., 2023; Premo & Kiel, 2016; Seddon et al., 2020) and are associated with youth internalizing problems (e.g., Cui et al.,^2020^; Lins et al., 2023).

However, highly influential models of child development contend that *both* dyad members actively shape the parent-child relationship and discourage reducing dynamic relationship influences to any one individual (e.g., Bronfenbrenner & Morris, 2006; Feldman, 2007). From childhood through adolescence, children increasingly influence parenting behavior (Avinun & Knafo, 2014) and contribute to dysfunctional interpersonal interactions (Conrad & Hammen, 1989; Hammen & Brennan, 2001). With respect to depression specifically, interpersonal models highlight stress generation, where individuals with elevated depressive symptoms experience greater stress due in part to dysfunctional interpersonal interactions (Hammen, 2006). Offspring of depressed mothers, especially daughters, are both more vulnerable to the adverse effects of negative interpersonal events *and* are more likely to elicit interpersonal stressors (e.g., reactive, unsupportive parenting by stressed parents; Hammen, 2009, 2012) that exacerbate this vulnerability. Especially among girls, elevated negative emotional reactivity and emotion regulation deficits elicit unsupportive emotion-related parenting (Kiff et al., 2011; Felton et al., 2021). Consistent with bidirectional models of stress generation that highlight the active role that children play in dysfunctional interpersonal interactions with depressed parents, unsupportive parenting among depressed mothers was specific to interactions with their distressed children, and did not generalize to their interactions with all their children (Conrad & Hammen, 1989). Drawing on seminal studies of stress generation in the context of maternal and adolescent depression, we sought to “zoom in” on dyadic processes that naturalistically reflect the landscape over which depression risk becomes entrenched for both parent and child.

## Biobehavioral synchrony

Dyadic processes during parent-child interactions include biobehavioral synchrony, or the moment-to-moment influences of rapid changes in each dyad member’s biobehavioral signals (e.g., affect, physiological functioning) on the other’s functioning (Feldman, 2007; Somers et al., 2023). Along with the rapid ebb and flow of affective changes during parent-child interactions, activity in the parasympathetic branch of the autonomic nervous system (ANS) is critical for flexible emotional responding and social engagement during social interactions (Porges, 2007; Thayer et al., 2012). In particular, when faced with an acute stressor, real-time withdrawal of the vagal brake (reflected in decreased respiratory sinus arrhythmia [RSA]) may facilitate recruitment of cardiometabolic resources needed for rapid, flexible context-appropriate socioemotional responding (Porges, 2007). Biobehavioral synchrony, including in affective and physiological domains, is thought to support smooth dyadic interactions (Davis et al., 2018; DePasquale, 2020; Feldman, 2007, 2012) and emerging offspring emotion regulation and socioemotional competence (Beeghly & Tronick, 2011; Beebe et al., 2010, 2012; Feldman, 2007, 2009, 2012; Tronick, 1989; Tronick & Beeghly, 2011). Within the context of challenging and potentially conflictual interactions, reciprocity and mutuality in parent-child interactions (e.g., shared positive affect) may facilitate cooperation (Kochanska & Aksan, 1995; Kochanska et al., 1995), including appropriate disclosure of disagreements and the ability to repair negative emotions associated with conflict. In turn, biobehavioral synchrony may provide an important foundation for the interpersonal regulation of negative emotional arousal that is central to the core emotional deficits in depression. Yet, the effects of parent-child biobehavioral synchrony, especially after infancy, have not been well-established (Lunkenheimer, 2024), and there are increasing concerns that synchrony may not be adaptive for older children in the context of elevated emotional risk (e.g., exposure to risky family/environmental contexts; DePasquale, 2020; Lunkenheimer, 2024).Yet, it remains unclear whether parental depressive symptoms and related parenting impairments (i.e., unsupportive ERSB) alter dyadic synchrony and/or modulate its effects on girls’ depression.

## Effects of synchrony in the context of parental depression and unsupportive parenting

Prior work suggests both parental depression and unsupportive parenting alter dyadic affective and physiological synchrony during parent-child interactions. Maternal depression is associated with negative affect synchrony in which each dyad member reciprocates the other’s negative affect (Connell et al., 2011; McMakin et al., 2011). For example, maternal depression (i.e., current depressive symptoms and diagnostic status) predicted greater durations of mutual negative affect and negative escalation of behavior with adolescent offspring across emotionally-salient tasks (including conflict discussion; Connell et al., 2011; McMakin et al., 2011). On the other hand, among dyads affected by maternal depression, there is typically a lack of synchrony or even divergence from each other’s positive affect (Kudinova et al., 2019; Morgan et al., 2020) and physiological regulatory (RSA) responses (Woody et al., 2016). Maternal depression history predicted less positive affect synchrony during positive emotion-eliciting tasks (Kudinova et al., 2019), even with preschoolers (Morgan et al., 2020) (although mothers who received treatment for prior depression may show greater positive affect synchrony; Vanwoerden et al., 2024). In addition, although maternal depression history was unrelated to children’s overall physiological response during conflict discussion, exposure to maternal depression was related to discordant patterns of RSA responses during a conflict discussion (Woody et al., 2016), highlighting that depression-related impairments were specific to *dyadic* functioning. Though less studied, similar effects have been demonstrated for greater negative parenting styles (e.g., emotional unavailability, psychological control), which were associated with discordant patterns of RSA responses (Han et al., 2019), whereas maternal supportive parenting was associated with less synchrony of negative emotions (Katz & Hunter, 2007) and greater synchrony in positive behaviors (Lunkenheimer et al., 2020).

However, it is unclear whether altered dyadic synchrony and depression-related unsupportive parenting independently or interactively increase risk for offspring depressive symptoms (Birk et al., 2022; DePasquale, 2020). Parent-child biobehavioral synchrony (i.e., where parents and their children match moment-to-moment changes in each other’s biobehavioral functioning) is hypothesized to support offspring emerging emotion regulation and socioemotional competence (for review, see Feldman, 2007). Supporting biobehavioral synchrony theory, there is compelling evidence that adolescent depressive symptoms are directly associated with attenuated positive affect synchrony (Kim et al., 2025); adolescent depressive symptoms are also associated with greater negative affect synchrony (Schwartz et al., 2012), especially in the context of anger (Bodner et al., 2018; Yap et al., 2010). By contrast, within the nascent literature on RSA synchrony, there is mixed evidence regarding the relation of RSA synchrony to youth mental health outcomes. For example, although RSA synchrony was negatively associated with significant preadolescent internalizing problems during child stress and parent-child conflict discussion Suveg et al., 2019), there is now mounting evidence for an alternate model where the effects of RSA synchrony on preadolescent youth mental health outcomes depend on parental mental health or parenting behaviors (Ahemaitijiang et al., 2021; Creavy et al., 2020; Oshri et al., 2023; West et al., 2020; Xu et al., 2024). Thus, understanding the interplay of parent ERSB and depressive symptom with parent–daughter synchrony is likely to clarify the implications of biobehavioral synchrony for girls’ depressive symptoms.

## Current Study Aims

This study evaluated a novel model where parent factors (depressive symptoms and unsupportive ERSB) and dyadic biobehavioral synchrony during conflict discussion affect preadolescent daughters’ depressive symptoms. To address knowledge gaps, the present study evaluated the putative effects of parent depressive symptoms and unsupportive ERSB on parent-daughter biobehavioral synchrony during naturalistic parent-daughter conflict discussions, and the interplay between unsupportive ERSB and biobehavioral synchrony on offspring depressive symptoms. This was specifically prosecuted among a sample of school-aged girls who possessed temperamental risk for affective problems and may be particularly sensitive to interpersonal risk processes for depressive symptoms (e.g., Dougherty et al., 2010; Hammen, 2009, 2012). Consistent with the principle of multifinality (Cicchetti & Rogosch, 1996), developmentally-informed designs prior to *acute* vulnerability among at-risk children are necessary to uncover pathways toward risk or resilience, which motivated the specific focus on girls in middle childhood (6–11 years), prior to the modal increase of depressive problems in adolescence. Thus, we adhered to developmental psychopathology principles, which explicitly consider biological sex, emotional risk, and developmental stage, to evaluate parenting factors and dyadic processes during conflict discussion that may increase at-risk youth’s depression vulnerability.

**Fig. 1:**
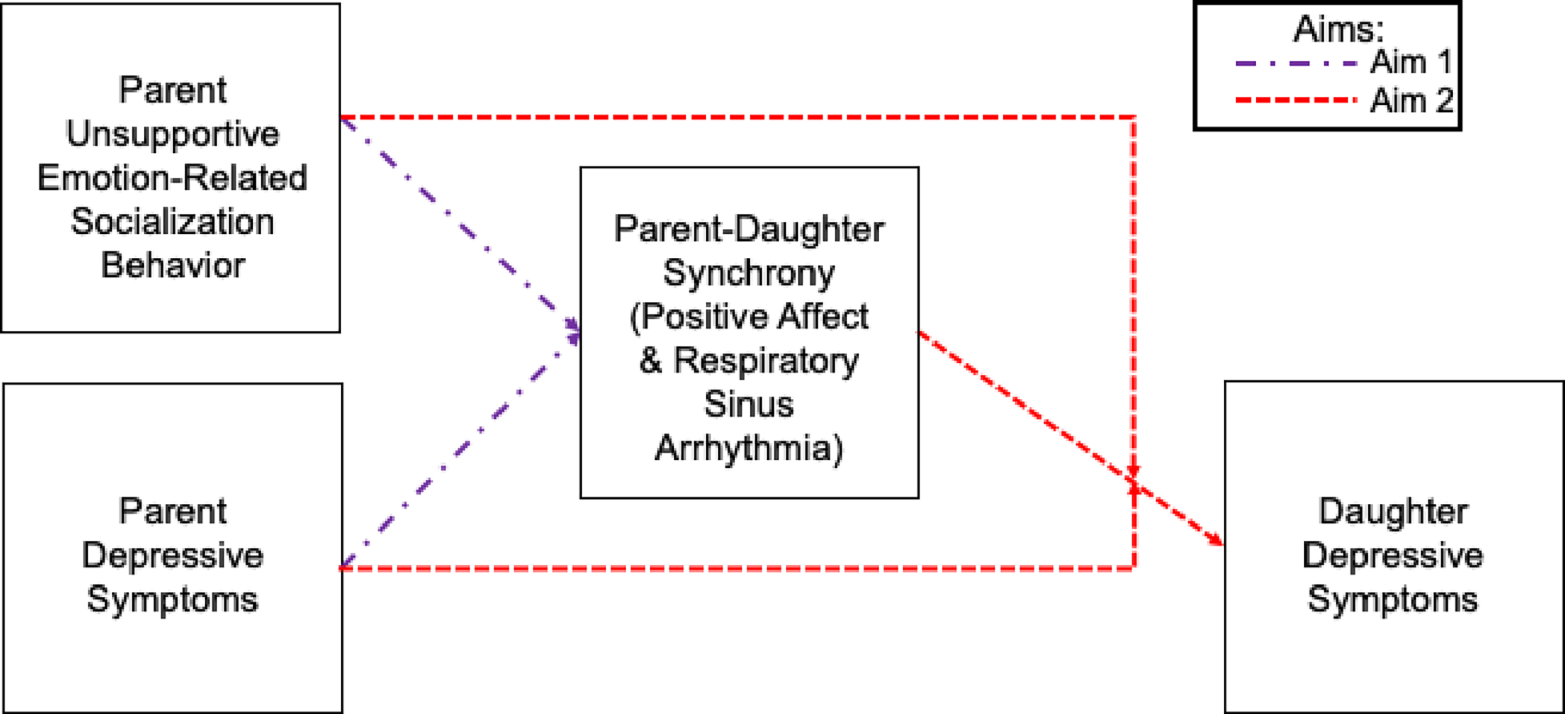
Conceptual model of the effects of parent factors on biobehavioral synchrony and the joint effects of parent factors and biobehavioral synchrony on preadolescent girls' depressive symptoms.

Drawing on biobehavioral synchrony theory (Feldman, 2007) and prior research (Connell et al., 2011; Kudinova et al., 2019; McMakin et al., 2011; Morgan et al., 2020), we hypothesized that parental depressive symptoms and unsupportive ERSB would each be inversely associated with (1a) positive affect synchrony. Further, informed by prior research (Han et al., 2019; Woody et al., 2016), we hypothesized that parental depressive symptoms and unsupportive ERSB would each be inversely associated with (1b) RSA synchrony. Our second aim examined (2a) the effects of dyadic positive affect and RSA synchrony on daughters’ depressive symptoms, including (2b) moderation by parental depressive symptoms or unsupportive ERSB. Given the underdeveloped literature, we did not have specific hypotheses for Aim 2. Our conceptual model is shown in Fig. 1.

## Method

### Participants

The enrolled sample included 80 parent-daughter dyads who participated in conflict discussions as part of an ongoing investigation of the cognitive, familial, and psychophysiological correlates of emotional development in school-aged girls, the [MASKED FOR REVIEW] Study. The [MASKED FOR REVIEW] Study involved one laboratory visit of four hours. Consistent with principles of NIMH Research Domain Criteria (RDoC), targeted recruitment efforts ensured sufficient range in girls’ negative emotionality without regard to a specific diagnostic taxon. Eligibility criteria included: (1) child whose sex assigned at birth is female; (2) child is between 6 and 11 years old during the time of screening, (3) fluency in English, (4) child must not have a history of seizures, seizure disorder, or other significant medical condition that would have prohibited full participation in study activities and (5) child must not have been previously diagnosed with intellectual disability. The [MASKED FOR REVIEW] IRB approved all study procedures prior to recruitment or data collection.

Data collection for the [MASKED FOR REVIEW] study began in February 2019 and was halted in March 2020 by the COVID-19 pandemic; data collection resumed May 2022 and completed September 2024. Girls were between 6 and 11 years of age (*M*_child age_ = 8.26 years, *SD*_child age_ = 1.51 years) and primarily participated with their biological/birth mothers (97.5% biological/birth parents; 87.5% female parents; *M*_parent age_ = 38.3 years, *SD* = 16.8 years). The children were racially diverse; 38.0% of children were identified by their parents as White, 13.9% were identified as Black, 7.6% were identified as Asian, and 40.5% were identified as multiracial. Approximately half (52.6%) of the sample reported average household incomes less than $150,000 per year, with a wide range from less than $25,000 to over $300,000 per year. Approximately one-third of the sample (36.3%) participated in the study prior to the COVID-19 pandemic. The pre-pandemic cohort did not differ from the post-pandemic cohort on any demographic characteristics, except the pre-pandemic cohort were less likely to have entered puberty, χ^2^(1) = 5.53, *p* = 0.024.

### Recruitment

Recruitment efforts targeted families with children who are “sad, angry, anxious, or irritable” or whose “emotions caused problems at school, home, or with peers.” To avoid known clinical and demographic biases associated with single-source recruitment sources (e.g., clinic-referred), participants were recruited diversely to the [MASKED FOR REVIEW] study from community settings, including pediatric offices, mental health service providers, tutoring centers, community/recreation centers, and local schools. Interested families contacted the research team and were carefully screened using a standardized script. If they satisfied eligibility criteria and remained interested in the study, families were then scheduled for a laboratory visit.

### Procedures

At the laboratory visit, parents provided informed consent and children assented to study procedures, which included laboratory-based parent-child interactions, rating scales, and diagnostic interviews. Parents rated common sources of conflict using the Issues Checklist (Prinz et al., 1979). Following widely-used procedures (e.g., Somers et al., 2024), during the conflict discussion task, an experimenter prioritized selecting a source of conflict that the parent rated the highest (M = 3.47 [SD = 0.95], Range: 1–5, on a scale where 1 = “calm”, 3 = “a little angry,” and 5 = “angry”) and as being unresolved, and instructed parents and their daughters to discuss this topic for 5 minutes (for more information, please see Supplementary Materials). The three most commonly-selected topics were cleaning up their bedroom (15%), fighting with siblings (15%), and screen time (13.8%). Participants were seated across from each other at a table and asked to remain seated and minimize movement during the discussion. Parents self-reported their depression and rated their daughters’ depression at the lab; in a few cases, due to time constraints, parents completed ratings at home and mailed the measures. Participants were compensated $100 for the laboratory visit.

### Measures

#### Parent and Child Depressive Symptoms

Parents reported on their depressive symptoms in the past six months using the 14-item *DSM-*oriented depressive problems subscale of the Adult Self-Report (ASR; Achenbach et al., 2003) using a 3-point scale from 0 to 2. Cronbach’s alpha, a conservative test of reliability, was 0.82. Primary analyses used the standardized T-score of parent depressive symptoms. 7.3% of parents reported borderline (T-score *≥* 60) depressive symptoms and 11.7% reported clinically-significant (T-score *≥* 65) depressive problems.

Parents reported on their child’s mental health problems in the past six months using the Child Behavior Checklist (CBCL; Achenbach & Rescorla, 2001). The 13-item *DSM-*oriented affective problems subscale (alpha = 0.53) uses a 3-point scale with higher scores reflecting elevated youth depressive symptoms. The DSM Affective Problems subscale was rationally-derived based on expert consensus, provides standardized scores based on national norms, and has extensive psychometric support (Berubé and Achenbach, 2006), including recent validation work in three independent datasets (Zelenina et al., 2025). Primary analyses used the standardized T-score of child depressive symptoms. 19.2% of children exhibited borderline (T-score *≥* 60) depressive problems and 20.5% exhibited clinically-significant (T-score *≥* 65) depressive problems. Child internalizing problems (alpha = 0.82) and total problems (alpha = 0.94) were also derived from the CBCL.

#### Observed Parent and Child Behavior

Trained researchers coded video-recorded conflict discussions for ERSB and parent and child affect in a series of three passes in which parent ERSB was coded first, followed by parent and then child affect. Coders were blind to participant characteristics. Coding training involved weekly meetings with an expert coder (the first author) on the coding manual and video review; coders were required to achieve interrater reliability (kappa > 0.65) with the first author on examples before coding videos independently; weekly meetings continued with the lead trainer to minimize drift. A randomly-selected 40% of videos were double-coded for reliability. Coding was conducted in the open-source software ELAN (ELAN, 2024) and moderate interrater reliability (time-unit kappa > 0.65; Altman, 1991; Bakeman et al., 2009) was achieved (average kappa on parent ERSB = 0.78; average parent affect kappa = 0.68; average child affect kappa = 0.75).

#### Parent Emotion-Related Socialization Behavior (ERSB)

 ERSB was coded using the Parent Emotion Socialization-Adolescent version (PESA; Martin & Hollenstein, unpublished) coding system. The PESA captures unsupportive parental ERSB in response to or about their child’s emotional expression and verbal statements, including dismissing (e.g., ignoring, changing the topic), mild rejection (e.g., minimizing, downplaying), and firm rejection (e.g., mocking, stonewalling). The PESA also includes three supportive codes. One ERSB code was applied to each speech act and then a second-by-second time series of parent ERSB was obtained. A total proportion of time spent engaged in unsupportive ERSB was obtained; however, this proportion was highly skewed and kurtotic (M = 0.01, SD = 0.03, Range: 0.00-0.20), and therefore, was dichotomized into whether any unsupportive ERSB was used or whether there was not any unsupportive ERSB.

#### Parent and Child Affect

Parent and child affect were coded using an adapted version of the Specific Affect Coding System (SPAFF5; Lougheed & Hollenstein, 2014) coding system. The SPAFF5 captures moment-to-moment changes in affect, following a multidimensional conceptualization of affect including changes in facial affect, vocal affect, body language, nonverbal behavior, and speech affect. One mutually exclusive affect code (positive/warm, dysphoric, angry/aggressive, or neutral affect) was applied to each 1-second epoch of the 5-minute conflict discussion. This resulted in approximately two 300-epoch long time series of parent and child affect; the number of codable epochs per conflict discussion ranged from 0 to 360 (M = 255, SD = 114). To mitigate potential bias when the majority of affect was unobservable, data from video recordings in which more than 50% of either parent or child affect was uncodable (*n* = 14) were treated as missing on affect synchrony. Uncodable epochs were largely due to camera angles and other sources of experimenter error, or participant mask or apparel that that obscured a participant’s face.

Limited variability in affect during the conflict discussion precluded time series analyses; instead, the total proportion of time spent engaged in shared positive affect was obtained, although this proportion was highly skewed and kurtotic (M = 0.01, SD = 0.02, Range: 0.00-0.14). Thus, positive affect synchrony was operationalized as whether the dyad exhibited any shared positive affect versus whether the dyad did not exhibit shared positive affect during the conflict discussion.

#### Parent and Daughter Respiratory Sinus Arrhythmia (RSA)

Disposable Ag/AgCl electrodes were placed on each participants’ chest in a modified Lead II placement, on the right clavicle, left clavicle, and lower right rib cage. Electrocardiogram (ECG) data were acquired using the Biopac MP160 system (Biopac Systems Inc., Goleta, CA) at a sampling rate of 2000 Hz. Coders used Mindware HRV Version 3.2.13 (Mindware Technologies, Inc., Gahanna, OH) to process the data; manually correct misidentified or unidentified R-peaks, such as ectopic beats due to physical movement; and obtain interbeat interval (IBI) data. Data were cleaned in 15-second epochs; no more than 10% of any epoch was edited for misidentified or unidentified R-peaks. For training on data cleaning, an expert graduate research assistant (the second author) initially cleaned RSA data from families from an independent training sample of parents and preadolescent daughters. During the training, research assistants reviewed a training manual and then collaboratively cleaned RSA data with the expert research assistant for one dyad and then cleaned RSA data from another family under the expert’s supervision. Next, the research assistants independently cleaned RSA data from four families (i.e., eight total individuals) in the training sample. Interrater reliability against the expert cleaner was checked; research assistants were required to achieve acceptable interrater reliability (average ICC > 0.70) before independent cleaning of ECG data from the families in the present study. Data cleaning was tiered, such that the trained research assistants conducted the initial data processing and cleaning of the files, then flagged segments of data for the expert research assistant to finalize in the case of complex cleaning decisions. The expert research assistant further randomly selected 20% of valid files for interrater reliability; the average interrater reliability was 0.93 in the present sample. Any discrepancies were discussed and resolved.

We estimated time-varying RSA for each five-minute discussion task using the MATLAB toolbox RSASeconds (Gates et al., 2015). Each of the cleaned IBI series from Mindware was interpolated at 4 Hz using a cubic spline to create equal data intervals. The data were then tapered using Peak Matched Multiple Windows (PM MW) and a short-time Fourier transform (STFT) was applied to moving 32-second IBI windows in order to obtain an estimate of the power spectrum for the 16th second of the window. Power estimates were obtained within the adult respiration frequency band (0.12–0.40 Hz; Berntson et al., 2007) for parent participants and within age-appropriate frequency bands for child participants (Shader et al., 2018). In short, the combination of PM MW and STFT produces point estimates of time-varying RSA for the central second in every rolling 32-second window, while drawing on information from the 16 s before and after the central second (Gates et al., 2015). Following recommendations from an experienced trainer at MindWare (Mindware, personal communication), any RSA values + 3 SD or more from the participant’s mean level of RSA during the task were removed (0.65% of child RSA data and 1.32% of parent RSA data). The PM MW/STFT method has been validated among adult and caregiver-child dyads (e.g., Gatzke-Kopp et al., 2022; Somers et al., 2021, Somers et al., 2021).

Each person’s RSA time series was first-differenced to remove linear trends in the data that may impact estimates of synchrony, resulting in estimates of reactive changes in RSA. We then estimated the cross-correlation (at a lag of 0) of parents’ reactive RSA estimates with their daughters’ RSA estimates; the resulting correlation coefficients were then Fisher transformed, which makes the correlation estimates (*z*-values) appropriate for statistical analyses (Gates et al., 2015). Estimates of RSA synchrony across the conflict discussion ranged from negative values (such that relative decreases in one dyad member’s RSA are associated with relative increases in the other’s RSA) to positive values (such that relative increases in one dyad member’s RSA are associated with relative increases in the other’s RSA).

#### Potential Covariates

Based on theoretical associations with parenting behavior and parent/youth depression (e.g., Daly, 2022; Oldehinkel et al., 2011), potential covariates included parent-reported child race and ethnicity, medication status, child age, pubertal status (0 = pre-pubertal, 1 = early puberty or more advanced stage; based on parent-report on the Pubertal Development Scale; Petersen et al., 1988), biological/birth parent status, parent gender, and annual household income. We also evaluated whether primary variables varied depending on the length of time between the lab visit and completion of questionnaires or differed among families who participated in the study before or after the COVID-19 pandemic restrictions were lifted.

### Data Analytic Plan

Primary analyses were conducted in a structural equation modeling framework in M*Plus* v. 8.11 using full information maximum likelihood estimation, a robust missing data technique (Enders, 2001). Maximum likelihood provides logistic regression coefficients for binary outcomes; numerical integration was also used to estimate these models. All models were fully saturated. All continuous predictors were grand mean-centered prior to analysis. Unstandardized estimates are reported. Significant interactions (*p* < 0.05) were evaluated at all levels of binary variables and at -1 SD, grand mean, and + 1 SD levels of moderators and independent variables. In addition, the regions of significance on continuous moderators were calculated using an online web utility (Preacher et al., 2006).

### Transparency and Openness

Our aims and hypotheses were preregistered (https://osf.io/dekf2). Deviations from our preregistration were motivated by characteristics of the data (e.g., limited variability in parents’ and children’s affect, particularly in negative affect and unsupportive behavior, which precluded assessment of negative affect synchrony and necessitated dichotomizing skewed positive affect and ERSB data; model convergence issues with strong autoregressive effects in RSA). Our sample size was determined according to our original analytic plan (described in our preregistration), which had power > 0.80 to detect desired effects (Schultzberg & Muthén, 2017). Due to participant privacy concerns, the raw data are not available; processed data are available from the first author upon reasonable request. We report how we determined our sample size, all data exclusions, all manipulations, and all measures in the study. The analytic code necessary to reproduce the primary analyses is available at https://osf.io/jw8qg/?view_only=cd86267ce0e74b34923512358c63dab5.

## Results

### Preliminary Analyses

Descriptive statistics for primary study variables are shown in Table 1. Values of RSA synchrony were skewed and kurtotic; however, when one outlier was removed, the resulting estimates of RSA synchrony were normally distributed. Thus, we removed the outlier (> 3.92 SD from the mean) on RSA synchrony from primary analyses. There were no other outliers on any primary study variables. Parent depressive symptoms were unrelated to unsupportive ERSB (Levene’s *t*(49.10) = 1.30, *p* = 0.20) and positive affect synchrony was similarly unrelated to RSA synchrony (*t*(40) = -1.18, *p* = 0.25). Based on their associations with levels of and/or missingness on primary study variables, child medication status, pubertal status, and pandemic status were included as covariates in all analyses. Additional information on covariate selection can be found in our Supplementary Materials.

**Table 1:** Descriptive statistics and associations for primary study variables

	M (SD) or % (Range)	*N*	1	2	3	4
1. Parent depressive symptoms	55.54 (7.64) (50–84)	69	---			
2. Parent unsupportive ERSB	42.50 (0–1)	73	0.93	---		
3. Positive affect synchrony	54.5 (0–1)	55	−0.74	1.30	---	
4. RSA synchrony	0.02 (0.21) (-0.34–0.85)	58	0.14	− 0.11	0.18	---
5. Child depressive symptoms	57.60 (6.77) (50–73)	78	0.29*	−0.63	−0.72	− 0.11

Parent and child depressive symptoms are reflected in ASR and CBCL T-scores, respectively. Associations between primary study variables were evaluated with bivariate correlations between continuous variables, *t*-tests between pairs of binary and continuous variables, and Chi-square tests between pairs of binary variables. * *p* < 0.05

### Primary Results

#### Aim 1. Associations between parents’ depressive symptoms and unsupportive ERSB on positive affect and RSA synchrony

To test Aim 1, we conducted a path model where positive affect and RSA synchrony were each predicted by parents’ depressive symptoms and unsupportive ERSB, after adjusting for child medication status, pubertal status, and pandemic status. Our results did not support Hypothesis 1a: Neither parent depressive symptoms, Est = 0.04, SE Est = 0.04, *p* = 0.41, nor parent unsupportive ERSB, Est = -0.86, SE Est = 0.61, *p* = 0.16, was associated with positive affect synchrony. Our results also did not support Hypothesis 1b: Neither parent depressive symptoms, Est = -0.00, SE Est = 0.00, *p* = 0.82, nor parent unsupportive ERSB, Est = -0.10, SE Est = 0.06, *p* = 0.13, predicted RSA synchrony. A similar pattern of results was obtained when affect and RSA synchrony were examined in separate models (see Supplementary Materials); thus, we present the results from a single combined model for parsimony.

Pandemic status was not associated with positive affect synchrony or RSA synchrony (all *p*’s *≥* 0.13). Child medication status was not associated with positive affect synchrony (*p* = 0.30) but was associated with lower RSA synchrony, Est = -0.14, SE Est = 0.06, *p* = 0.010. Child pubertal status was not associated with positive affect synchrony (*p* = 0.42) but was associated with greater RSA synchrony, Est = 0.20, SE Est = 0.06, *p* < 0.001.

#### Aim 2. Associations between positive affect and RSA synchrony on daughters’ behavioral problems and whether these associations are modulated by parent depressive symptoms or unsupportive ERSB

To test Aim 2, two path models^1^ were conducted where daughters’ depressive symptoms were predicted by synchrony (positive affect or RSA), parents’ depressive symptoms and unsupportive ERSB, and the interaction effects of synchrony and unsupportive ERSB, after adjusting for covariates (child medication status, pubertal status, and pandemic status).

#### Effects of positive affect synchrony and parent factors on daughters’ depressive symptoms

With respect to aim 2a, there was not a main effect of parent depressive symptoms, Est = 0.46, SE Est = 0.28, *p* = 0.10, or positive affect synchrony, Est = -2.83, SE Est = 2.52, *p* = 0.26, on daughters’ depressive symptoms. Similarly, with respect to Aim 2b, the interaction effect between positive affect synchrony and parent depressive symptoms was not significant, Est = -0.13, SE Est = 0.32, *p* = 0.69. However, there was a significant interaction effect between positive affect synchrony and the presence of unsupportive parent ERSB on daughters’ depressive symptoms, Est = 8.45, SE Est = 3.63, *p* = 0.020. Covariate effects of child medication status, pubertal status, and pandemic status were not significant (all *p*’s > 0.40).

**Fig. 2:**
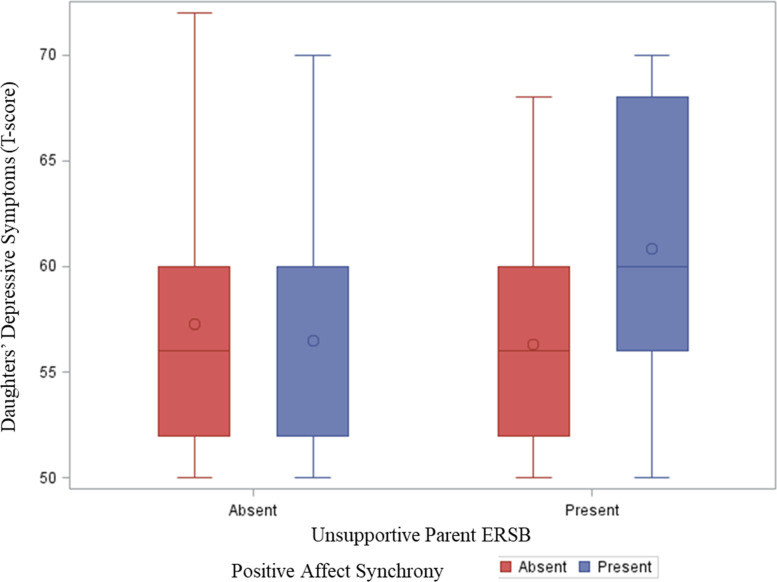
Associations between parent unsupportive emotion-related socialization behavior (ERSB) and daughters’ depressive symptoms, by positive affect synchrony

Post-hoc probing revealed the effect of parent unsupportive ERSB on daughters’ depressive symptoms was significant during positive affect synchrony (54.5% of the sample; Est = 5.45, SE Est = 2.28, *p* = 0.017) but not significant in the absence of positive affect synchrony, Est = -3.00, SE Est = 2.65, *p* = 0.26 (see Fig. 2). There was a significant effect of positive affect synchrony on daughters’ depression in the presence of unsupportive parent ERSB, Est = 5.62, SE Est = 2.52, *p* = 0.026. However, there was no significant effect of positive affect synchrony on daughters’ depressive symptoms without unsupportive parent ERSB, Est = -2.83, SE Est = 2.52, *p* = 0.26.

#### Effects of RSA synchrony and parent factors on daughters’ depressive symptoms

With respect to Aim 2a, neither parent depressive symptoms, Est = 0.16, SE Est = 0.11, *p* = 0.14, nor unsupportive parent ERSB, Est = 1.22, SE Est = 1.62, *p* = 0.45, predicted daughters’ depressive symptoms. However, with respect to Aim 2b, there was a significant RSA synchrony x parent depression effect on daughters’ depressive symptoms, Est = -2.37, SE Est = 0.82, *p* = 0.004. The interaction effect between unsupportive parent ERSB and RSA synchrony was not significant, Est = 5.73, SE Est = 9.59, *p* = 0.55. Covariate effects of child medication status, pubertal status, and pandemic status were not significant (all *p*’s > 0.28).

**Fig. 3:**
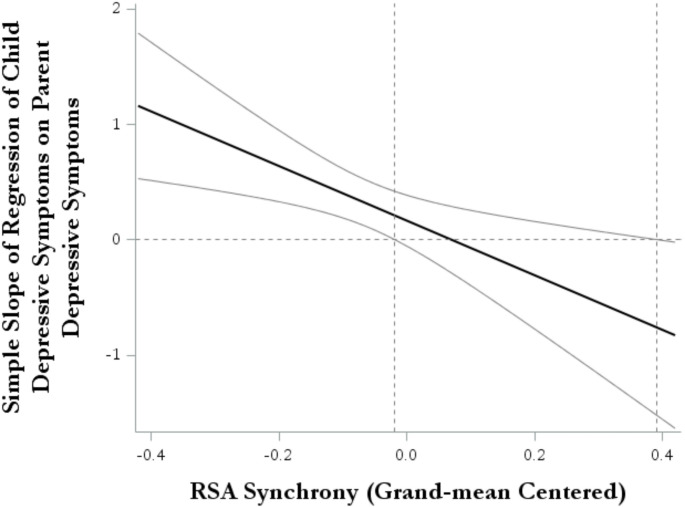
Interaction Effects Between RSA Synchrony and Parent Depressive Symptoms on Daughters’ Depressive Symptoms

Post-hoc probing of the RSA synchrony x parent depressive symptoms interaction revealed that the effect of parents’ depressive symptoms on daughters’ depressive symptoms was not significant at high (+ 1 SD) (representing strong synchrony), Est = -0.33, SE Est = 0.25, *p* = 0.19, or average levels (representing mild synchrony) of RSA synchrony, Est = 0.18, SE Est = 0.13, *p* = 0.16. However, at low (-1 SD) levels of RSA synchrony (representing strong asynchrony or divergence in physiological responses), parents’ depressive symptoms were positively associated with daughters’ depressive symptoms, Est = 0.68, SE Est = 0.19, *p* < 0.001. Analysis of the regions of significance (interpreted within 2 SD of the mean of RSA synchrony; Roisman et al., 2012) indicated that at approximately the mean or below (46.6% of the sample, who exhibited asynchrony or divergence in physiological responses) levels of RSA synchrony, there was a positive association between parents’ depressive symptoms and daughters’ depressive symptoms, Est = 0.21, SE Est = 0.10, *p* = 0.05, whereas at greater levels of RSA synchrony, there was no association between parents’ and daughters’ depressive symptoms. Taken together, these results suggest that at below average levels of RSA synchrony, there was a positive association between parents’ and daughters’ depressive symptoms, whereas at average or greater levels of RSA synchrony, there was no association between parents’ and daughters’ depressive symptoms (see Fig. 3).

The effect of RSA synchrony on daughters’ depressive symptoms was significant at + 1 SD levels of parent depression (Est = -25.96, SE Est = 10.06, *p* = 0.010). However, there were no significant effects of RSA synchrony on daughters’ depressive symptoms at average (Est = -8.03, SE Est = 6.30, *p* = 0.20) or low (-1 SD) levels of parent depressive symptoms, Est = 9.89, SE Est = 7.71, *p* = 0.20.

#### Specificity of results to daughters’ depressive problems relative to internalizing or total problems

We also conducted four additional path models to evaluate the independent and interactive effects of parent factors (depressive symptoms and unsupportive ERSB) and synchrony (positive affect synchrony and RSA synchrony) on daughters’ internalizing problems and total behavior problems, adjusting for covariates (see Supplementary Materials for full model results). Dyadic synchrony did not directly predict girls’ internalizing or total problems, nor did it interact with parent factors to predict girls’ internalizing or total problems (all *p*’s > 0.05).

## Discussion

Using observational and physiological data collected among temperamentally at-risk school-aged girls and their parents, the current study examined pathways by which parent factors (depressive symptoms and parenting behavior) and dyadic processes influence preadolescent girls’ depression risk. Results provided mixed support for hypotheses but revealed the consequences of parent-daughter biobehavioral synchrony processes depend on the interpersonal context. Girls’ elevated negative emotional reactivity and emotion regulation deficits may confer risk for future adolescent depression specifically through entrenched bidirectional processes that precipitate, maintain, and reinforce depressive symptoms (e.g., Hammen, 2009, 2012). Yet, the effects of parent-child biobehavioral synchrony after infancy have not been well-established (Lunkenheimer, 2024). Contrary to expectations, among families where parents engaged in unsupportive emotion-related socialization behavior, youth had greater depressive symptoms if they also exhibited positive affect synchrony with their parents. By contrast, in the context of elevated parent depressive symptoms, greater levels of parent-daughter respiratory sinus arrhythmia (RSA) synchrony were associated with fewer child depressive symptoms. Taken together, results underscore that the broader interpersonal context shapes the effects of bidirectional processes of biobehavioral synchrony on girls’ depressive symptoms. Further, results were specific to girls’ depressive symptoms (versus internalizing or total behavior problems) and thus reaffirm the primacy of the interpersonal context in girls’ depression.

### Associations of interpersonal contextual factors with biobehavioral synchrony

Contrary to hypotheses, parent depressive symptoms and unsupportive ERSB were unrelated to parent-daughter positive affect and RSA synchrony. Whereas maternal depression status previously accounted for differences in positive affect synchrony during positive emotion-eliciting tasks (Kudinova et al., 2019; Morgan et al., 2020), parent depressive symptoms did not account for differences in the presence of shared positive affect during a conflict discussion. One possible explanation is that depression-related impairments in positive affect synchrony may be most pronounced during interpersonal contexts with more frequent positive affect. For example, supporting the context-specificity of depression-related alterations in positive affect, Kudinova et al. (2019) observed that mother-preadolescent dyads with a history of maternal depression exhibited reduced positive affect synchrony during a pleasant vacation planning discussion, but not during a conflict discussion. It is also important to note that 20% of parents in the present sample endorsed current subclinical or clinically-significant depressive symptoms, whereas depression-related impairments in positive affect synchrony were observed among families with and without histories of maternal depression (Kudinova et al., 2019; Morgan et al., 2020). Further, parents with varying levels of depression symptoms may have different experiences in outpatient treatment, which positively predicted positive affect synchrony (Vanwoerden et al., 2024). Future work is needed to evaluate the effects of both parent depression history and current symptoms levels on positive affect synchrony across different emotionally-salient contexts and in samples specifically recruited for varying levels of parent depression.

Prior work with clinically and sociodemographically at-risk samples has demonstrated that maternal depression history and symptoms were negatively associated with RSA synchrony during challenging tasks, including conflict discussions (Suveg et al., 2019; Woody et al., 2016). However, null associations between ANS (either RSA or IBI) synchrony during conflict discussion and maternal depression (West et al., 2020) and parent emotion dysregulation (Ahemaitijiang et al., 2021) have also been reported. Whereas parent-child IBI synchrony during conflict resolution was negatively associated with unsupportive parenting (e.g., psychological control; Han et al., 2019) in some studies, RSA synchrony during conflict resolution was unrelated to a wide range of parenting behaviors in other samples (Ahemaitijiang et al., 2021; Oshri et al., 2023; Xu et al., 2024). Taken together, these results suggest that global aspects of parent emotion socialization in middle childhood, including the broader household emotional climate and specific emotion-related parenting behaviors, may operate independent of moment-to-moment processes that unfold during conflict discussion. However, as emotional risk becomes more acute (e.g., in the case of clinical maternal depression, Woody et al., 2016, or poverty, Suveg et al., 2019), parent factors may affect dynamic interaction processes more acutely. This persuasively suggests that prospective designs are needed to elucidate when and for whom contextual factors affect moment-to-moment physiological synchrony.

### Interplay between interpersonal contextual factors and biobehavioral synchrony on girls’ depressive symptoms

Results also suggest that interpersonal contextual factors and parent-daughter biobehavioral synchrony jointly contribute to girls’ depressive symptoms. Children’s perception and interception of positive affect synchrony may differ depending on the broader context in which it occurs. In contrast to prior evidence that positive affect synchrony was *negatively* associated with adolescent depressive symptoms (Kim et al., 2025), results here suggest that shared positive affect was *positively* associated with preadolescent girls’ depressive symptoms in the presence of unsupportive parent ERSB. Further, this pattern of results was specific to girls’ depressive symptoms; positive affect synchrony was marginally *negatively* associated with total behavior problems. In the context of unsupportive ERSB, children may perceive shared positive affect as inauthentic (e.g., laughing at the child rather than laughing with the child), which may increase risk for depression (Theran, 2011). Another possibility is that shared positive affect may reflect genuinely reciprocal dyadic interactions that promote a warm tone (e.g., playful sarcastic banter) but hinder problem-solving and successful resolution of conflict, especially in the context of parent behavior that dismisses or invalidates the child’s perspective. By contrast, shared positive affect, in the absence of harsh or rejecting behavior, during conflict may reflect children’s internalization of parental prosociality and willingness to cooperate with parents during disagreements (Kochanska & Aksan, 1995; Kochanska et al., 1995), which protect against a broad range of behavioral concerns, highlighting the importance of adaptive parent-child conflict resolution during this developmental stage. To our knowledge, this is the first study to consider parental factors x positive affect synchrony interactions on youth depressive symptoms, and results warrant replication in future work that employs interaction tasks that elicit a wide range of affect expressions and parenting behavior (e.g., in vivo challenging tasks).

These results also contribute to a burgeoning literature that suggests physiological synchrony moderates the associations of parent factors on youth mental health problems. Among a sample of temperamentally at-risk school-aged girls and their parents, the adverse effect of parent depressive symptoms on daughters’ depressive symptoms was specific to dyads who exhibited RSA asynchrony and to depression risk rather than broader internalizing or overall behavior problems. Put another way, RSA synchrony during parent-daughter conflict discussion buffered against parent depression-related risk for youth depressive symptoms. These results diverge from prior evidence that ANS synchrony during a conflict discussion was associated with more child behavior problems when mothers exhibit greater depressive symptoms (West et al., 2020) and with more child aggressive behavior when parents exhibit greater emotional dysregulation (Ahemaitijiang et al., 2021). Notably, these prior studies evaluated RSA synchrony on slower time scales (e.g., 30-second epochs; West et al., 2020), whereas the present study evaluated synchrony in second-by-second fluctuations in RSA, closer to the temporal resolution on which RSA is thought to change during social interactions (Porges, 2007; Thayer et al., 2012). Following theoretical advances in parent-infant interpersonal dynamics (i.e., “match-then-calm”; Wass et al., 2024), adaptive relationship processes may differ depending on the timescale on which they are assessed, such that in-phase second-by-second RSA synchrony may reflect matching of children’s emotional arousal whereas anti-phase synchrony in subsequent responding may reflect parents’ calming of children’s emotional arousal (e.g., PNS activation in response to children’s PNS withdrawal). Research is needed that evaluates whether the independent and interactive effects between parental factors and physiological synchrony differ depending on the timescale on which synchrony is assessed. This could also explain why, unlike prior work (e.g., Ahemaitijiang et al., 2021; Oshri et al., 2023; Xu et al., 2024), the parenting behavior x RSA synchrony interaction was unrelated to child outcomes. Further, results demonstrate that the interplay between parent-daughter synchrony and contextual influences is specific to youth depression, rather than broad internalizing or overall problems, underscoring the critical role of the interpersonal environment in early symptom differentiation and the emergence of depressive problems.

### Strengths and limitations

As depression rates in young girls rise, these preliminary findings provide important implications in further understanding interpersonal factors that influence depressive symptoms in middle childhood. To our knowledge, this is the largest sample of parent-daughter dyads in which biobehavioral synchrony and its interplay with parent mental health and ERSB has been evaluated; this is critical as gender-specific models of emotion expression affect parents’ ERSBs and youth expectations for parenting (Chaplin et al., 2005; Fivush, 1989; Klimes-Dougan et al., 2007; Waslin et al., 2023). In addition, the present study is one of few multimodal studies to simultaneously assess affect and physiological synchrony (DePasquale, 2020). Evaluation of multiple unique domains of dyadic synchrony (i.e., positive affect, physiology) more comprehensively ascertains individual differences in interpersonal dynamics across key domains (emotional, arousal/regulatory, and social systems; Somers et al., 2023) and varying levels of conscious awareness and outward expression. Further, positive affect and RSA synchrony were not significantly correlated, suggesting that these processes may operate independently during conflict discussions in middle childhood, rather than as a part of an underlying phenomena of biobehavioral synchrony that is evident in infancy (Feldman, 2007) and early childhood (e.g., Lunkenheimer et al., 2021). The present investigation was also advantaged by assessment of physiological synchrony and observed parent unsupportive ERSB, which circumvents possible shared method and informant biases associated with traditional parent-report measures, and can capture behavioral cues that parents may not notice or deem significant but may have cumulative effects on their daughters’ well-being.

The results of this study should also be interpreted in the context of its limitations. Although this study recruited girls in middle childhood (i.e., 6 to 11 years) and statistically adjusted for current pubertal status, developmental stage and pubertal status may moderate the effects of parent and dyadic factors on youth depressive symptoms. As emotional awareness improves across middle childhood (Gnepp et al., 1987; Larsen et al., 2007), younger children in the study may have responded differently to parents’ unsupportive ERSB or real-time changes in parents’ biobehavioral functioning than the older children (although our preliminary analyses did not suggest age-related differences in primary study variables). Inclusion of fathers allowed for examination of dyadic relationships beyond mother and child; whereas parent gender was not related to any primary study variable, including positive affect and RSA synchrony, insufficient numbers of father-daughter dyads precluded testing patterns differently in mothers and fathers. In addition, while the study benefited from multimodal assessment of synchrony, changes in RSA synchrony may be due to dyadic coordination of internal (e.g., vagal influences on the heart) or external (e.g., respiratory changes that influence RSA) processes, which the present study is unable to directly assess. Likewise, despite the use of multiple methods across multiple units of analysis, parents’ report of conflict topics and intensity and of both their own and their child’s depressive symptoms may have been subject to shared informant bias, and parents and children may differ in their perceptions of parent-child interactions and youth internalizing problems (Zelenina et al., 2025). Parent ratings of their child’s depressive symptoms also had relatively lower internal consistency, which may reflect heterogeneity in children’s depressive symptoms (e.g., sleep and energy-related disturbances versus affective disturbances). Given that unsupportive parenting (in contrast to supportive parenting) transmits the intergenerational effects of maternal depression (e.g., Lins et al., 2023; Lovejoy et al., 2000; Seddon et al., 2020), we focused specifically on observed unsupportive parenting; however, limited variability in unsupportive ERSBs and affect during the conflict discussion, which is common in laboratory-based studies (e.g., Capaldi et al., 1994; Hollenstein et al., 2004; Snyder et al., 2003), necessitated the dichotomization of our observed affect and behavioral variables and precluded assessment of associations with negative affect synchrony. Microcoding systems captured the presence or absence of discrete changes in affect and ERSBs; however, relative to global coding approaches, microcoding approaches may be less sensitive to differences in the intensity of affective behavior and unsupportive ERSBs. Our focus on parent ERSB was aligned with our focus on dyadic processes involved in youth depression risk but may not have captured other parenting behaviors associated with depression risk (e.g., withdrawal). As parents in this study typically endorsed relatively moderate levels of conflict and low levels of depressive symptoms, results may not generalize to “hotter” conflict discussions and other interaction tasks that elicit greater frequency of negative affect (e.g., in vivo stressors; Suveg et al., 2019) or to families that face higher risk due to more acute parent mental health problems.

### Future directions

These findings offer several directions for future research. Surprisingly, we failed to observe associations between parent depressive symptoms and unsupportive ERSB. Although the present study benefited from the use of a reliable observational assessment of unsupportive ERSB, children may have perceived parents’ behavior differently than our coders; evaluating child perception of parental socialization behavior could be an important area of future investigation. Our investigation focused on parent-daughter dyads given gender-specific models of emotion socialization; our results require replication and warrant future evaluation of gender differences in ERSB and its interplay with biobehavioral synchrony among sufficiently-powered samples. Additionally, although the present study was not powered to detect three-way interactions, the interplay between synchrony and parent factors on youth outcomes may depend on overall levels of individual biobehavioral functioning (e.g., average level of RSA; Connell et al., 2011). Across multiple levels of analysis (e.g., positive affect, PNS-mediated RSA), concurrent synchrony modulated the association of parental depressive symptoms and unsupportive parenting with daughters’ depressive symptoms. Analyses operationalized synchrony as second-by-second matching of positive affect states or RSA reactivity across a conflict discussion. Whereas our work is consistent with the extant literature that has predominantly focused on concurrent synchrony (e.g., Miller et al., 2023, for meta-analysis), assessment of time-lagged synchrony would enable evaluation of the drivers of synchrony, which may further illuminate mechanisms of risk transmission (Somers et al., 2023). Further, theoretical advances consider synchrony as a dynamic construct that changes over the course of an interaction (Gordon et al., 2024). Future research is needed that evaluates the dynamic nature of synchrony; although parenting was not associated with overall levels of synchrony, unsupportive parent ERSB may disrupt synchrony during moments when parents engage in such behavior, and in turn, these parenting-related momentary ruptures in synchrony may confer emotional risk to youth if they are not repaired.

### Conclusion and Clinical Implications

School-aged girls, and especially daughters of parents with depression, are at heightened risk for depression, which directly influences lifelong health and functioning. Considering that risk for depression and conflictual relationships with parents increases precipitously during the transition to adolescence (Steinberg & Morris, 2001), middle childhood is a sensitive period to intervene in parent-daughter interaction dynamics. Yet, family processes, including bidirectional influences between parents and daughters, are not explicitly targeted in interventions for childhood depression (Kovacs, 2023), which predominantly target individual-level psychological or biological factors (Hankin & Griffith, 2023). Family-focused approaches may enhance the effectiveness of youth depression treatment and protect against relapse, warranting the need to develop novel approaches for integrating parents into youth depression treatment (Weisz et al., 2023). Results of the present study elucidate interpersonal dynamics involved in school-aged daughters’ depressive symptoms that may serve as novel intervention targets. Here, the interplay between parent factors and dyadic biobehavioral synchrony predicted daughters’ depressive symptoms, though important differences emerged for physiological synchrony versus positive affect synchrony. Pending replication, results suggest that interventions that simultaneously reduce unsupportive parent ERSB and promote parent-daughter physiological synchrony and positive affect synchrony may mitigate against the early emergence of girls’ depressive symptoms.

## Supplementary Information

Below is the link to the electronic supplementary material.

Supplementary Material 1

## Data Availability

Due to participant privacy concerns, the raw data are not available; processed data are available from the first author upon reasonable request. We report how we determined our sample size, all data exclusions, all manipulations, and all measures in the study. The analytic code necessary to reproduce the primary analyses is available at https://osf.io/jw8qg/?view_only=cd86267ce0e74b34923512358c63dab5.

## Footnotes

1 Separate path models were conducted as we did not have sufficient degrees of freedom to estimate all interaction effects in one model.
