# Supplementary material for: The Dyadic Context of Preadolescent Girls’ Depressive Symptoms: Elucidating The Role of Biobehavioral Synchrony
Source: J Child Fam Stud. 2026 Apr 10;35(5):1178–95. doi: 10.1007/s10826-026-03300-6 (PMC13156177; doi:10.1007/s10826-026-03300-6)
Supplement: Supplementary file 1 — Supplementary Material 1 [file 10826_2026_3300_MOESM1_ESM.docx]

**Supplementary Materials**

**Conflict discussion ratings.** Following widely-used procedures (e.g., Somers et al., 2024), during the conflict discussion task, an experimenter prioritized selecting a source of conflict that the parent rated the highest and as being unresolved, and instructed parents and their daughters to discuss this topic for 5 minutes. On average, parent-report of conflict intensity was 3.47 (SD = 0.95), on a scale where 1 = “calm”, 3 = "a little angry,” and 5 = “angry.” One parent (1.3%) reported the conflict as “calm,” nine parents (11.3%) reported the conflict as a 2, 31 parents (38.8%) reported the conflict as “ a little angry,” 22 parents (27.5%) reported the conflict as a 4, and 12 parents (15.0%) reported the conflict as “angry.” Conflict ratings were missing for 5 (6.3%) parents.

**Missingness on primary study variables.** Out of the 80 families who were enrolled in the study, missingness on primary study variables ranged from 2.5% to 26.3%. Out of the 80 families who were enrolled in the study, seven families did not consent to audio/video recording and thus did not have any coded data. Four families did not have usable recordings (e.g., due to experimenter error or technical failures) for dyadic affect coding. Additionally, of the 69 families who had dyadic positive affect data, to mitigate potential bias when the majority of affect was unobservable, data from video recordings in which more than 50% of either parent or child affect was uncodable (*n* = 14) were treated as missing on affect synchrony but were still included in RSA synchrony analyses. Of the 80 families who enrolled in the study, 15 parents and 16 children did not have physiological data. Two of these families did not consent to wearing physiological equipment, 13 parents and 13 children had psychophysiological files that could not be processed due to equipment failure or poor data quality (i.e., excessive movement artifact, signal loss), and 1 child’s data was removed due to a reported heart murmur. Combined, this resulted in our total of 16 children and 15 adults who were missing physiological data prior to estimating time-varying RSA in MATLAB (see Figure 1). One outlier on RSA synchrony was removed from the analytic sample. Any dyads in which one person was missing RSA data were missing on RSA synchrony. Missingness on parent and child depressive symptoms was due to failures to return the ASR and CBCL following the lab visit, respectively.

| Table 1. Total sample size for each primary study measure, for the enrolled sample and final analytic sample (total N = 80) | | |
| --- | --- | --- |
|  | Enrolled sample (N) | Analytic sample (N) |
| Parent depressive symptoms | 69 | 69 |
| Parent unsupportive ERSB | 73 | 73 |
| Positive affect synchrony | 69 | 55 |
| RSA Synchrony | 59 | 58 |
| Child depressive symptoms | 78 | 78 |

Figure 1. Flow chart of missing data.


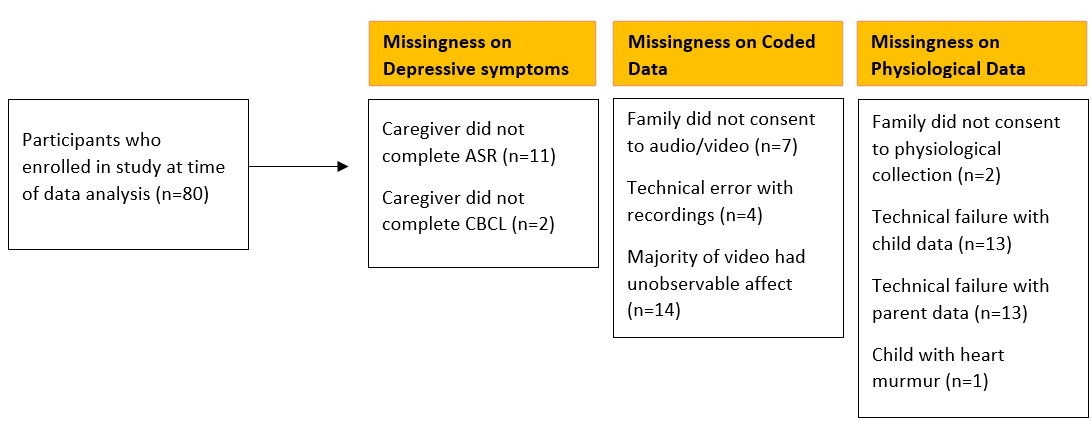


**Covariate selection.** Child medication (e.g., allergy, stimulant medication) status was associated with RSA synchrony, *t*(55) = 2.34, *p* = 0.023, such that children who took medication had lower synchrony. Child pubertal status was also associated with RSA synchrony, *t*(51) = -3.024, *p* = .004, such that girls who were in or advanced past early puberty had lower RSA synchrony (M = -0.08, SD = 0.16) than girls who were pre-pubertal (M = 0.09, SD = 0.23). Pandemic status was associated with parent unsupportive ERSB, χ²(1) = 3.83, *p* = 0.044, such that parents who participated pre-pandemic were more likely to show unsupportive ERSB. Pandemic status was also related to missingness on parent depressive symptoms, χ²(1) = 4.76, *p* = 0.047, child depressive symptoms, χ²(1) = 4.36, *p* = 0.036, and positive affect synchrony, χ²(1) = 4.16, *p* = 0.045, such that dyads who participated post-pandemic were more likely to be missing on parent and child depressive symptoms and positive affect synchrony. No covariates were associated with levels of or missingness on parent supportive ERSB (all *p*’s > 0.05). Thus, child medication status, pubertal status, and pandemic status were included as covariates in all analyses.

**Aim 1 Analyses**. Results of models that examined Aim 1 hypotheses in separate models for each outcome yielded similar results to the primary model that examined both outcomes simultaneously (see Table 2).

| Table 2. Results of Aim 1 models when synchrony outcomes were modeled separately | | | | | | |
| --- | --- | --- | --- | --- | --- | --- |
|  | Outcome: Positive Affect Synchrony | | | Outcome: RSA Synchrony | | |
|  | Est | SE | *p*-value | Est | SE | *p*-value |
| Unsupportive parent ERSB | -0.876 | 0.611 | 0.152 | -0.096 | 0.063 | 0.129 |
| Parent depressive symptoms | 0.035 | 0.042 | 0.413 | -0.001 | 0.003 | 0.842 |
| Child medication status | -0.696 | 0.665 | 0.295 | -0.143 | 0.056 | 0.010 |
| Pre-pandemic status | 1.073 | 0.687 | 0.118 | 0.075 | 0.058 | 0.197 |
| Child pubertal status | 0.630 | 0.715 | 0.378 | 0.196 | 0.055 | <.001 |

**Specificity of results to daughters’ depressive problems relative to internalizing or total problems.** We explored whether (3a) the effects of parent depressive symptoms, unsupportive ERSB, and (3b) their interaction were specific to girls’ depression or were also evident in girls’ internalizing or total behavior problems. Four additional path models evaluated the independent and interactive effects of parents’ depressive symptoms and unsupportive ERSB on daughters’ internalizing problems and total behavior problems, adjusting for covariates.

**Effects of positive affect synchrony and parent factors on girls’ internalizing and total problems.** With respect to 3a & 3b, in models predicting girls’ internalizing problems (T-score), neither the main effects of parent depressive symptoms, *p* = 0.08, parent unsupportive ERSB, *p* = 0.30, or positive affect synchrony, *p* = 0.40, or the interaction effects between positive affect synchrony and parent depressive symptoms, *p* = 0.56, or the interaction effect between positive affect synchrony and parent unsupportive ERSB, *p* = 0.08, were statistically significant predictors of girls’ internalizing problems. Covariate effects of child medication status, pubertal status, and pandemic status were also not significant (all *p*’s > 0.49).

Similarly, in models predicting girls’ total behavior problems (T-score), parent depressive symptoms, *p* = 0.06, and parent unsupportive ERSB, *p* = 0.27, were not statistically significant predictors of girls’ total behavior problems. Positive affect synchrony, Est = -6.83, SE Est = 3.37, *p* = 0.04, was a significant predictor of girls’ total behavior problems. Neither the positive affect synchrony x parent depressive symptoms interaction, *p* = 0.60, nor the positive affect x parent unsupportive ERSB, *p* = 0.06, interaction effects were statistically significant. Covariate effects of child medication status, pubertal status, and pandemic status were not significant (all *p*’s > 0.68).

**Effects of RSA synchrony and parent factors on daughters’ internalizing and total behavior problems.** In models predicting girls’ internalizing problems (T-score), there was a (3a) main effect of parents’ depressive symptoms, Est = 0.75, SE Est = 0.27, *p* = 0.006, but not of unsupportive parenting, *p* = 0.49, or of RSA synchrony, *p* = 0.26. In addition, neither (3b) the interaction effect between RSA synchrony and parents’ depressive symptoms, *p* = 0.79, nor the interaction effect between RSA synchrony and parents’ unsupportive ERSB, *p* = 0.95, were statistically significant predictors of girls’ internalizing problems. Covariate effects of child medication status, pubertal status, and pandemic status were not significant (all *p*’s > 0.71).

Likewise, in models predicting girls’ total behavior problems (T-score), there was a (3a) main effect of parents’ depressive symptoms, Est = 0.77, SE Est = 0.25, *p* = 0.002, but not of parent unsupportive ERSB, *p* = 0.54, or RSA synchrony, *p* = 0.82. In addition, neither (3b) the interaction effect between RSA synchrony and parents’ depressive symptoms, *p* = 0.35, nor the interaction effect between RSA synchrony and parents’ unsupportive ERSB, *p* = 0.96, were statistically significant predictors of girls’ total behavior problems. Covariate effects of child medication status, pubertal status, and pandemic status were not significant (all *p*’s > 0.56).
